# Pathogenic Fungi—Structural Biology and Taxonomy

**DOI:** 10.3201/eid1104.041291

**Published:** 2005-04

**Authors:** Errol Reiss

**Affiliations:** * Centers for Disease Control and Prevention, Atlanta, Georgia, USA

**Keywords:** Pathogenic Fungi, Structural Biology, Taxonomy, Book Review

Structural Biology and Taxonomy is the first volume in a series authored by leading medical mycologists. The series' scope is to review progress in basic research on zoopathogenic fungi, a timely effort as medical mycology moves into the genomics era. General knowledge of fungal pathogens is assumed. This volume begins by discussing the cell wall which, besides its roles in pathogenesis, is now a practical drug target. The molecular architecture of fungal walls remains elusive because of difficulty in correlating chemical composition with the ultrastructural layers and uncertainty concerning the linkages connecting major cell wall polymers: glucans, mannan, and chitin.

The basis for morphogenesis is the holy grail of medical mycology because temperature-sensitive dimorphism is a stratagem used by several deep-seated fungal pathogens. The fungal cell cyle is considered with respect to the mechanism of sequential gene expression in *Candida albicans*, since little is known about the cell cycle in pathogenic molds. Important interpretation is provided about the hyphal form of *C. albicans*, which clarifies the germ tube's role in morphogenesis and, potentially, in disease.

The molecular genetics of morphogenesis in *C. albicans* follows. Hyphal growth during infection is arguably a pathogenic factor since it thwarts phagocytosis. Genes controlling hyphal development include ones that are upregulated during cell elongation and adherence to epithelia. Fine tuning of morphogenesis is illustrated by the "enhanced filamentous growth" gene which, when knocked out, blocks the transition to the mycelial form. Early steps in this transition are complex, with at least 2 signaling pathways identified: 1 stops yeast growth and another, with a heat shock protein 70-type profile, initiates the assembly of proteins necessary for mycelial growth.

As the focus on morphogenesis continues, dimorphism in several endemic mycoses is concisely reviewed. Heat shock proteins are emphasized because of the temperature-sensitive morphogenesis to the tissue form. Yeast-form–specific genes identified in *Histoplasma capsulatum* function in calcium/calmodulin signaling pathways and sulphur metabolism. Calcium-dependent signaling pathways and heat shock protein expression regulate dimorphism in *Paracoccidioides brasiliensis* and have broad implications for other pathogens. *Coccidioides immitis* dimorphism involves the construction and rupture of the spherule, or tissue form, for which chitin and glucan synthetases and hydrolases are key enzymes. A further chapter discusses how surface membrane G-protein receptors in *C. albicans* and other fungi transmit external stimuli through 2 major protein kinase cascades. These govern multiple functions, including hyphal development and the secretion of pathogenic factors.

Departing from the morphogenesis theme, chapters on phylogenetic analysis help clarify the taxonomy of noncultivatable medical fungi (excluding *Pneumocystis*). The evolution and population genetics of 3 agents of major deep-seated mycoses, which follow, are of more general interest. Finally, in a return to the original cell wall theme, the fine structure of mannans and galactomannans is explained as a useful adjunct in classifying fungi.

Pathogenic Fungi—Structural Biology and Taxonomy is an important interpretation of recent literature, a valuable addition to collections, and recommended reading for investigators seeking a broad appreciation for the current state of the art.

